# Sonomechanomyography (SMMG): Mapping of Skeletal Muscle Motion Onset during Contraction Using Ultrafast Ultrasound Imaging and Multiple Motion Sensors

**DOI:** 10.3390/s20195513

**Published:** 2020-09-26

**Authors:** Yan To Ling, Christina Zong-Hao Ma, Queenie Tsung Kwan Shea, Yong-Ping Zheng

**Affiliations:** Department of Biomedical Engineering, The Hong Kong Polytechnic University, Hung Hom, Hong Kong, China; jane.yt.ling@connect.polyu.hk (Y.T.L.); czh.ma@polyu.edu.hk (C.Z.-H.M.); queenie.tk.shea@connect.polyu.hk (Q.T.K.S.)

**Keywords:** muscle motion onset, ultrafast ultrasound, electromyography (EMG), mechanomyography (MMG), maximum voluntary contraction (MVC)

## Abstract

Background: Available methods for studying muscle dynamics, including electromyography (EMG), mechanomyography (MMG) and M-mode ultrasound, have limitations in terms of spatial resolution. Methods: This study developed a novel method/protocol of two-dimensional mapping of muscle motion onset using ultrafast ultrasound imaging, i.e., sono-mechano-myo-graphy (SMMG). The developed method was compared with the EMG, MMG and force outputs of tibialis anterior (TA) muscle during ankle dorsiflexion at different percentages of maximum voluntary contraction (MVC) force in healthy young adults. Results: Significant differences between all pairwise comparisons of onsets were identified, except between SMMG and MMG. The EMG onset significantly led SMMG, MMG and force onsets by 40.0 ± 1.7 ms (*p* < 0.001), 43.1 ± 5.2 ms (*p* < 0.005) and 73.0 ± 4.5 ms (*p* < 0.001), respectively. Muscle motion also started earlier at the middle aponeurosis than skin surface and deeper regions when viewed longitudinally (*p* < 0.001). No significant effect of force level on onset delay was found. Conclusions: This study introduced and evaluated a new method/protocol, SMMG, for studying muscle dynamics and demonstrated its feasibility for muscle contraction onset research. This novel technology can potentially provide new insights for future studies of neuromuscular diseases, such as multiple sclerosis and muscular dystrophy.

## 1. Introduction

**Figure 1:**
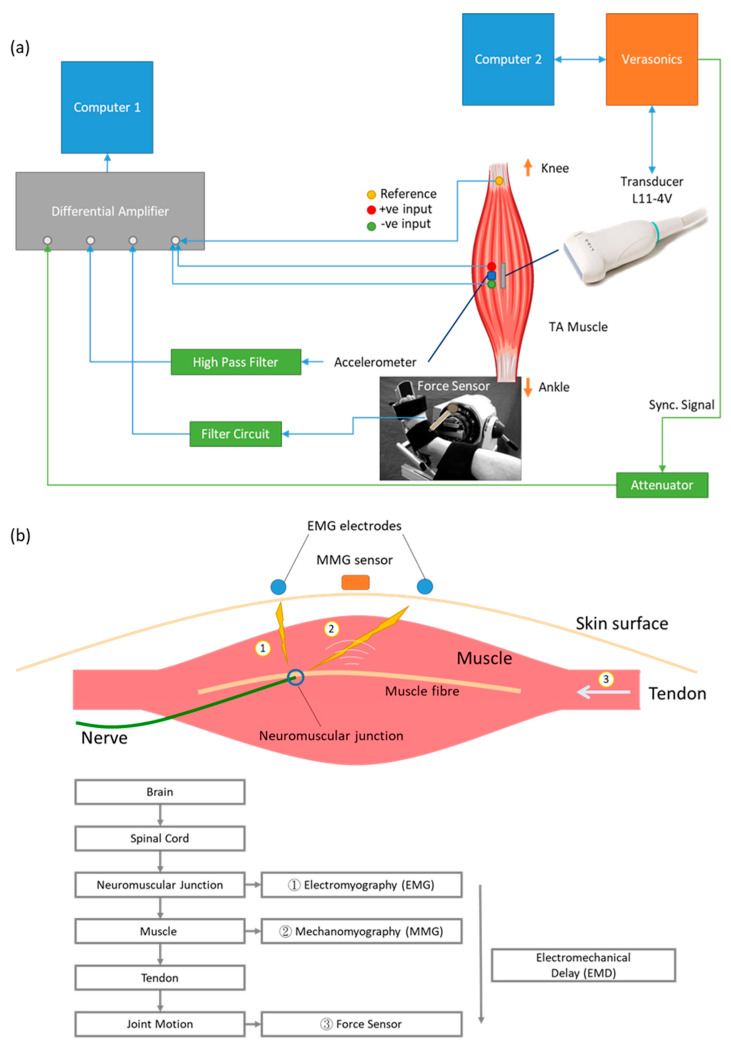
(**a**) Signal transmission of a voluntary muscle contraction from brain to muscle. Action potential reaches the neuromuscular junction and is detected by EMG electrodes. The action potential triggers muscle fiber motion, which is detected by MMG sensor. Muscle motion then generates joint motion through pulling the tendon. Joint motion can be detected with force sensor. The time difference between onset of EMG and onset of force is electromechanical delay. (**b**) Block diagram of setup. One computer was used to record EMG, MMG and force data from differential amplifier. Another computer was connected with the Verasonics system and controlled the transmission and recording of the ultrasound probe. A synchronization signal was generated by the Verasonics system, lengthened by an arbitrary function generator and recorded by the differential amplifier.

Multiple bio-signals can be detected using different sensing modalities during the contraction of a skeletal muscle (Figure 1a). From the initial idea generated in the brain neurologically to the exertion of muscle force biomechanically, the generation of human motion involves a sequence of events. When action potential passes through the motor neurons and reaches the neuromuscular junction, electrical activities can be detected using either surface or needle electromyography (EMG). When muscle fibers start to contract and the resulting vibration propagates to the skin surface, these tiny vibrations can be detected by surface mechanomyography (MMG). Muscle fiber motion then pulls tendons to generate joint motion and force which can be measured by load cell, pressure sensor and force sensor [1,2,3]. Although arrays of EMG and MMG were developed for two-dimensional measurements in previous studies [4,5], they were unfortunately limited only to those bio-signals that could propagate to the skin surface. It has been, however, difficult to show the spatial variation within the muscle with existing technologies and testing protocols.

The elapsed time between onset of the EMG signal and force output is defined as electromechanical delay (EMD) [2]. EMD has been used as an indicator for tracking the normal electrophysiological events and also as a reliable variable indicator for some pathologies such as spastic cerebral palsy [6] and ligament laxity [7]. However, depending on methodological design, the EMD has been shown to have huge inter-individual temporal variations ranging from 45 to 120 ms [1]. Many attributions can be put forward to reduce this variation, including adipose tissue thickness [8], skin fold thickness affecting MMG response [9], location of the sensors [9] and muscle stiffness [3]. To match the tissue motion onset from inside the muscle together with the onset of the signals collecting from outside the muscle on the skin surface, efforts have been made by adopting ultrafast ultrasonography [2,3].

Ultrasound imaging is acquired based on the reflection of sound waves in tissues and can be used for monitoring muscle architectural change during contraction in real time using a technique called sonomyography (SMG) [10,11]. SMG uses an ultrasound frame rate of around 30 frame/s and is thus not able to capture the fast changes inside the muscle, such as muscle motion onset. As muscle structure changes during contraction, the intensity of the reflected sound wave also changes, which can be detected by ultrafast ultrasound imaging, with frame rates larger than 1000 frame/s. This is how the onset of muscle spatial motion can be observed in the image by detecting the intensity change in the ultrasound image. Ultrasound imaging technologies have been used to study skeletal muscle contraction characteristics [12], neuromuscular disorders and biomechanical material properties [13].

In terms of onset timing, Vasseljen et al. (2006) showed that the muscle onset detected by ultrasound had a delay and was generally after that of EMG in the back muscle of lumbar multifidus during contraction [14]. Meanwhile, a later study by the same group reported that the onset difference between intramuscular EMG and concurrent M-mode ultrasound in abdominal muscles varied. Specifically, the EMG onset was detected before the ultrasound onset in obliquus externus abdominis, but such a pattern was found to be reversed in transversus abdominis and obliquus internus abdominis [15]. This group suggested correcting for such muscle-dependent onset time difference, which was assumed to be a measurement artifact [15]. However, EMG measures the electrical event, while ultrasound imaging detects the mechanical event of muscle contraction. It is reasonable for the onset time measured by these two different methods to be different from each other. Meanwhile, other studies showed consistent delay from EMG to ultrasound onset and suggested that this time delay could be used for functional assessment of muscle [2,3]. Such inconsistency of muscle onset measured by different sensing modalities in the existing literature implies that this topic requires further study.

In terms of spatial variation, Hug et al. (2011) showed that there was no significant difference between the muscle fascicle motion and the myotendinous junction motion in repeatedly stimulated bicep contraction [16]. However, the recording of images at the muscle and at the tendon was not synchronous in this study, which may have given rise to uncertainties in the results. Dieterich et al. (2017) showed dependence of onset time with regard to the depth within the muscle upon visual analysis [17]. However, this study did not quantify the result with reference to the muscle structure. Deffieux et al. (2006) demonstrated the detection of axial tissue displacement and velocity in biceps under stimulated contraction using ultrafast ultrasound imaging at 5000 frames per second (fps) [18] but without the detection of muscle movement concerning both depth and width of a muscle region.

Recent muscle onset-related research mainly focused on onset determination algorithms [19,20,21] and application of the techniques in patients [22] or in athletes [23]. This research concerns mostly EMG recordings.

The two-dimensional (2D) spatial variation of muscle fiber onset remains a gap in our knowledge. As the action potential (electro-chemical event) propagates through the muscle fibers, it is expected that the propagation time along this direction generates a mechanical wave with a certain time delay. While the action potential travels away from the innervation zone, where a nerve enters the muscle itself, it is expected to observe a timely changing onset of the generated mechanical wave upon proceeding the action potential. Hence, depicting the onset of the propagating wave (tissue velocity—mechanical event) from more than one location within a muscle in accordance with conduction velocity (electro-chemical event) from electromyography (EMG) has not been investigated so far and merits further study.

This paper introduces a newly developed system for simultaneous recording of surface EMG, MMG, force and ultrafast ultrasound imaging and reports the findings of the time delays between onset of electro-chemical event and mechanical events in relation to force output, while using external sensors including surface EMG, MMG and force in combination with ultrafast ultrasound signals detected from inside the muscle, during step-by-step increases in muscle activity. This study adopts a new method of onset detection for ultrafast ultrasound muscle imaging and identifies the pattern of 2D muscle onset observed in ultrafast ultrasound imaging with reference to anatomical musculoskeletal structure changes. We hereby define the new term sonomechanomyography (SMMG), i.e., sono-mechano-myo-graphy, as using ultrafast ultrasound to depict the mechanical vibration generated in muscle motion.

## 2. Materials and Methods

The first part of this study investigated how muscle onset measured from EMG, MMG, force and ultrafast ultrasound differed at different strengths of voluntary contractions. To observe the spatial variation of muscle onset measured by ultrafast ultrasound imaging, the second part of this study focused on the muscle motion onset measured in longitudinal and transverse views of the muscle. The findings are reported separately in this paper.

### 2.1. Subjects

A convenience sampling approach was adopted to recruit 14 healthy subjects aged between 18 and 35 years. Subjects were required not to have undergone any surgical intervention on their right tibialis anterior and the related bones, namely tibia, cuneiform and metatarsal. They were required not to have history of any metabolic, neurologic or orthopedic disease or sequel preserved at the time of investigation. All participants were asked to sign a written informed consent document before the experiment. While subjects demonstrating occasional sports engagement or sedentary lifestyles were included, professional sports players were excluded from this study. Human ethics approval was obtained from the Human Subjects Ethics Sub-Committee (HSESC) of the Hong Kong Polytechnic University (HSESC Reference Number: HSEARS20170716001).

In the first part of the study, the data from 7 (out of 10) subjects (3M + 4F, aged 25.9 ± 1.8 years, height of 171 ± 12 cm, and weight of 68.7 ± 12.1 kg) with a total of 72 trials were available for muscle onset analysis. In the second part of study, the data from 8 (out of 10) subjects (5M + 3F, aged 25.6 ± 1.7 years, height of 172 ± 11 cm, and weight of 66.2 ± 14.0 kg) with a total of 20 trials (11 in transverse view and 9 in longitudinal view) were available for muscle onset analysis. 

### 2.2. System Setup

Figure 1b shows the system diagram of the setup for simultaneous measurement of EMG, MMG, force and ultrafast ultrasound. The system consisted of two computers for control and data acquisition. For the ultrasound imaging machine, the Vantage 128 (Verasonics, Kirkland, WA, USA) was used with the probe L11-4v. The frame rate was set at 20 kHz with an imaging depth of 3.5 cm. A total of 14,000 frames were recorded in each trial. An 8-channel differential amplifier (RM6280, Cheng Yi, China) with a gain of 2000 was used to collect EMG, and the MMG and force data were collected using the same system. The sampling frequency was set to 20 kHz to match the sampling frequency of the ultrasound system. The arbitrary function generator (AFG3021, Tektronix, Beaverton, OR, USA) received an external trigger from the Verasonics and gave a single pulse output as synchronization signal to the differential amplifier. A three-axis accelerometer module (GY-61 DXL335, Analog Devices, Norwood, MA, USA) was used as sensor for surface MMG. Only the motion perpendicular to skin surface was recorded. Button electrodes (Sichiray, China) were used to collect EMG signals from skin. The electrodes on the proximal and distal sides of the accelerometer were connected to the positive and negative inputs of the differential amplifier, respectively. The reference electrode was put on the head of fibula. A force sensor (FlexiForce A201, Tekscan, South Boston, MA, USA) was used to measure the force exerted by the subject’s isometric contraction of TA muscle.

### 2.3. Experimental Procedure

In the first experiment (Experiment 1), the subject was first asked to sit onto a dynamometer chair (Humac/Norm Testing and Rehabilitation System, Computer Sports Medicine, Inc., Stoughton, MA, USA). After adjustment of the chair, subject was asked to perform a few TA isometric contractions such that the boundaries of the muscle could be marked on the skin. Measurement of muscle length and leg circumference was conducted before placement of EMG, MMG and ultrasound probes. The subject was instructed to perform contraction a few times to check that all signals could be properly recorded. In order to normalize the EMG values, it was necessary to determine the EMG at the maximum voluntary contraction (MVC) level as a reference value. The subjects were asked to perform contraction at their maximum force for three times. The arithmetic mean of the amplitude of the highest signal portion with 3000 ms fixed duration was defined as MVC level. After a rest of around 5 min, the subject performed contraction of 10%, 20%, 30%, 40% and 50% MVC in a randomized sequence when they heard an audio cue (a click sound). Each MVC level contained three trials for each subject. Visual feedback of the force generated during each contraction was provided in real time to guide the subject to reach the required contraction level. There was a resting time of 5 min between each trial. The subject was detached from the dynamometer chair after completion of the 15 trials, when the data collection was complete. 

For the second experiment (Experiment 2), a similar protocol was followed and conducted on subjects, but with ultrafast ultrasound imaging recording only. Subjects were asked to contract at their maximum force. Ultrasound images were taken at longitudinal view (where depth of image is the anterior–posterior position and width of image is proximal–distal position) and transverse view (where depth of image is the anterior–posterior position and width of image is medial–lateral position) for 2 trials each.

### 2.4. Data Analysis for Experiment 1

**Figure 2:**
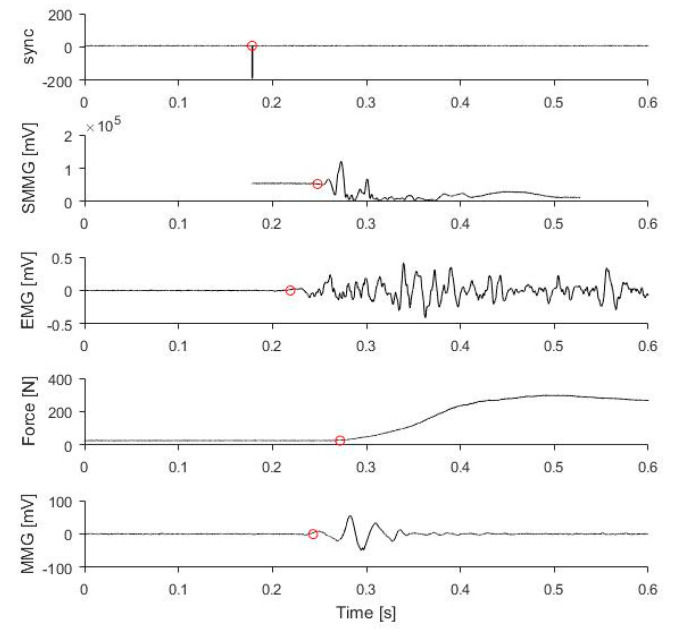
Onset detection in different signals. (Top to bottom) Synchronization signal, filtered ultrasound intensity variation in a pixel (SMMG), EMG, force, MMG signals plotted against time. The red circles mark the points of onset.

The MATLAB program (MATLAB 2016a, The MathWorks, Inc., Natick, MA, USA) was used for image reconstruction using algorithms provided by the Verasonics. After image reconstruction, 1000 Hz low pass filter was applied to the time-varying B-mode intensity signal at each pixel (Figure 2). For each trial, an initial period of resting state was selected as baseline. The standard deviation of the period was calculated. The onset time was determined as the time point at which the signal first rose above or fell below five times the standard deviation (SD) from the baseline value. The threshold of 5SD was determined from a pilot study, and it worked best when compared to the SD of less than five times during the pilot study. Trials with baseline drift were excluded. For each trial, the mean onset at the central 1 cm × 1 cm of the recorded image was calculated as the representative onset time.

A band-pass filter of 50 Hz to 4000 Hz was applied to both the EMG and MMG signals. The standard deviations of the signals at baseline were calculated. The first time point when the signal went above or below five times the standard deviation was considered as the onset time of that signal. A low-pass filter of 1000 Hz was applied to the force signal. The first time point when the signal went above five times the standard deviation was considered as the onset time of force signal. The time point at which a falling edge was detected in synchronization signal was regarded as the time for Verasonics to start recording. An example of onset detection of the above signals is shown in Figure 2.

Trials with onset of EMG which was found before the synchronization signal (i.e., subject contracted before hearing the audio cue) were excluded from analysis. For a valid subject test, at least 10 valid trials were required in total and more than 1 trial at each MVC level. Otherwise, they would be further excluded from the analysis. Averaged onset time in each physiological measurement for each MVC level was calculated for each subject. 

Statistical analysis was done using SPSS Statistics (SPSS Statistics 25, IBM, New York, NY, USA). Two-way analysis of variance (ANOVA) was performed to compare the onset time between different physiological measurements and MVC levels. Further analysis was conducted to quantify the differences between different positions within the imaged area. The imaged area was separated into 25 regions of interest (ROI) in 5-by-5 grids of equal area. The mean onset time for each ROI in each trial was calculated. The mean for each MVC level of each subject was then calculated by averaging the 25 ROIs. Three-way ANOVA was performed to determine the variation of onset time with respect to the MVC level and the location within muscle. 

### 2.5. Data Analysis for Experiment 2

The ultrasound image processing was similar to that in Experiment 1, except that the 1000 Hz low-pass filter was changed to a 50–1000 Hz band-pass filter. The area of tibia and skin in the transverse view were manually removed from data analysis. The ultrasound images and the onset maps (25 ROIs) were then averaged. The variation in onset time along and perpendicular to the middle aponeurosis was then computed. To study the significance of the trend, independent *t*-test was performed to compare the onset time at each consecutive 5 mm distance. 

## 3. Results

### 3.1. Onset Time of Different Physiological Measurements and MVCs

**Figure 3:**
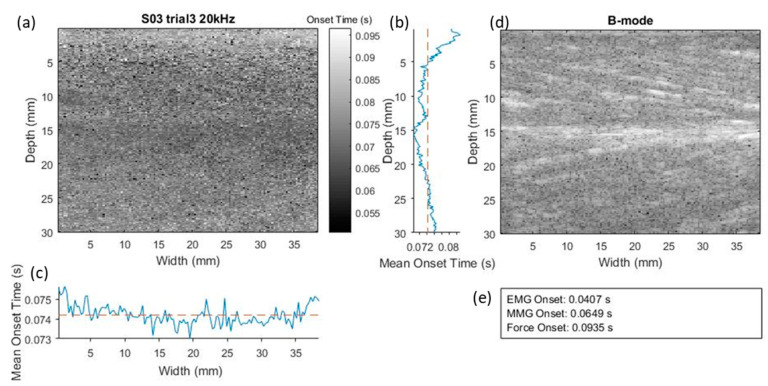
As example illustrating the ultrasound SMMG onset map (**a**), B-mode ultrasound image of muscle along longitudinal view (**d**) and the EMG, MMG, force onset time (**e**) recorded in a trial. In the mean onset time plots, the blue solid line represents the mean onset time measured using SMMG along the muscle depth direction (**b**) and longitudinal direction (**c**), while the orange dotted line represents the overall mean onset time measured with SMMG.

Figure 3 shows an example of SMMG onset map, the B-mode image and the EMG, MMG and force onset time recorded in a trial. The mean onset time along the muscle depth and longitudinal directions are plotted next to the map. In this trial, SMMG onset fell between the MMG onset and the force onset.

**Figure 4:**
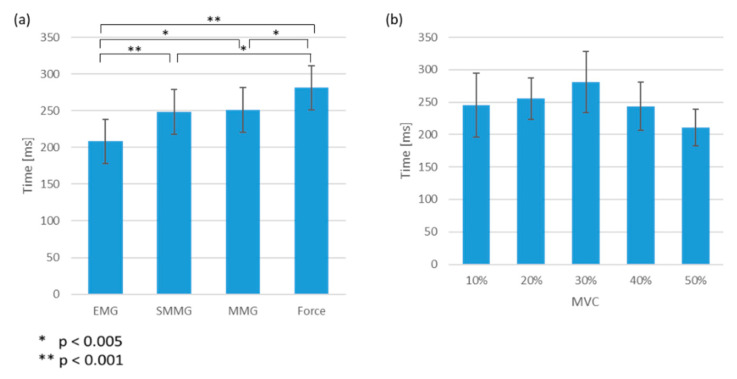
(**a**) Time of onset in EMG, SMMG, MMG and force signals from the audio instruction. Error bar showing standard error. (**b**) Time of onset of measured with SMMG at 10%, 20%, 30%, 40% and 50% MVC levels from the audio instruction. Error bar showing standard error.

No significant interaction effect between the measurement method and the MVC levels was observed. Pairwise comparisons showed significant differences between all pairs of onset among different physiological measurements, except between SMMG and MMG (Figure 4a). The EMG onset led SMMG, MMG and force onset by 40.0 ± 1.7 ms (*p* < 0.001), 43.1 ± 5.2 ms (*p* < 0.005) and 73.0 ± 4.5 ms (*p* < 0.001), respectively (Figure 4a). No significant difference was found for the onset time measured by ultrasound imaging between different MVC levels Figure 4b.

### 3.2. Spatial Variation in Ultrasound Onset

**Figure 5:**
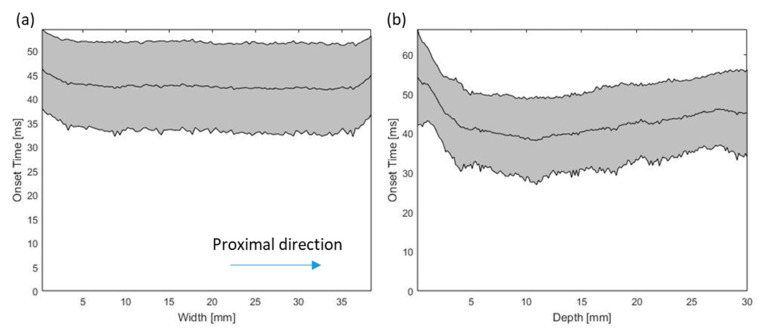
Mean onset time at different (**a**) widths and (**b**) depths of ultrasound image in longitudinal view. Grey area represents plus and minus one standard deviation at each position.

The mean onset times of all trials at different locations along the muscle depth and longitudinal directions are shown in Figure 5. No significant interaction effect between the positions and the MVC levels was observed. No significant difference was found among different MVC levels. The results of pairwise comparisons are shown in Table 1 and Table 2. There was a significant difference between onset time at different depths in muscle, but not in the other dimensions.

**Figure 6:**
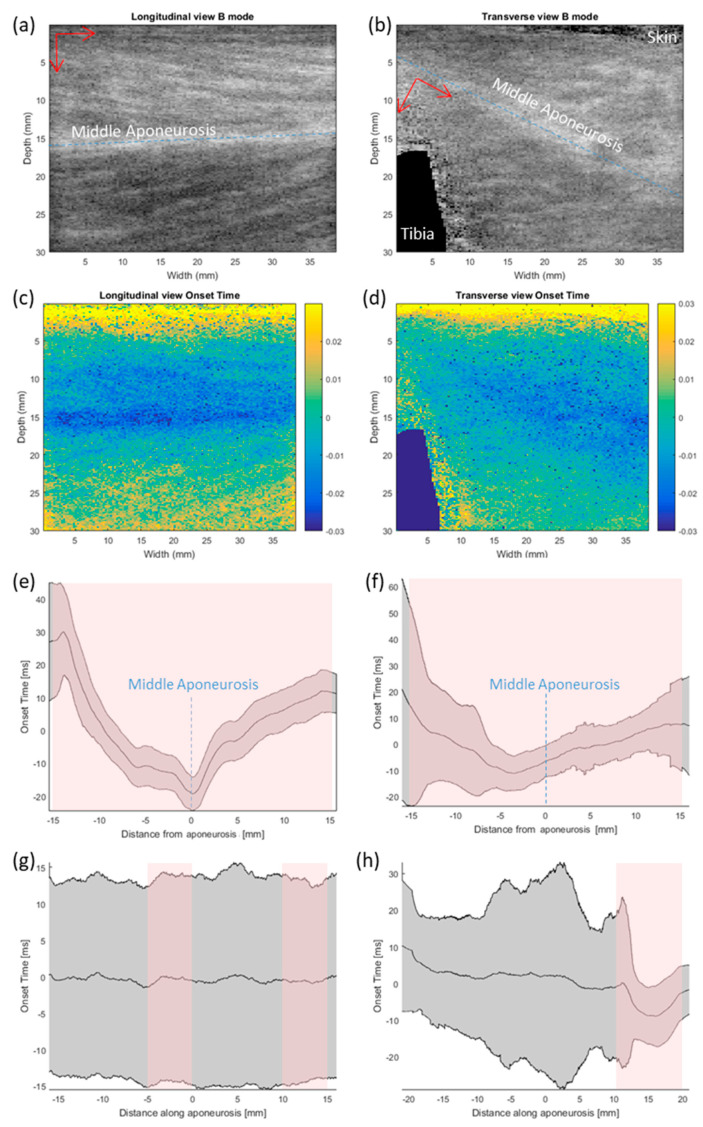
Muscle onset imaged in longitudinal view and transverse view. (**a**,**b**) show the averaged B-mode image in longitudinal view and transverse view. Blue dotted line indicates the midline of the middle aponeurosis. Red arrows mark the direction of distance taken to form charts (**e**–**h**). (**c**,**d**) show the corresponding onset time, with zero in time marking the mean of the area imaged. (**e**,**f**) show the variation in onset time against distance from the middle aponeurosis. The location of aponeurosis is marked in blue dotted line. (**g**,**h**) show the variation in onset time against distance along the middle aponeurosis. Grey area represents plus and minus one standard deviation at each position. Pink shaded areas denote significant trend at 5 mm intervals (*p* < 0.05, independent *t*-test).

Figure 6a–d shows the B-mode image and the corresponding map of ultrasound-measured onset in longitudinal and transverse view. In the longitudinal view Figure 6e, it is clearly shown that muscle motion started earlier at the middle aponeurosis and then spread out towards skin surface and deeper regions (*p* < 0.001 for comparison between each 5 mm distance). In the transverse view (Figure 6f), however, the earliest motion was at 4 mm from the aponeurosis on the superficial side that was closer to the skin (*p* < 0.005 for comparison between each 5 mm distance). There was not much variation in the other directions (Figure 6g–h).

## 4. Discussion

This study developed a new system for simultaneous and synchronized recording of surface EMG, MMG, force and ultrafast ultrasound imaging (SMMG); we applied this system to identify the sequential time delays between onset of electro-chemical event (detected by EMG) and mechanical events in relation to force output (detected by MMG and force sensor) and further integrated the ultrafast ultrasound imaging to detect the signal depicted from inside the muscle during step-by-step increases in muscle activity. Several interesting findings related to the onset were observed in this study.

### 4.1. Feasibility of Using Ultrafast Ultrasound Imaging to Map the Onset Inside Muscle 

This study has demonstrated the feasibility of using ultrafast ultrasound imaging to study the onset mapping inside the muscle, which is so far the first study looking into this issue to the best of our knowledge. We defined this new signal about muscle motion as sonomechanomyography (SMMG). With reference to the anatomical musculoskeletal structures, this study observed the pattern of 2D muscle onset from the ultrafast ultrasound image during TA muscle contraction in static position. Specifically, it appears that muscle motion started earlier at the middle aponeurosis and then spread out towards the skin surface and deeper regions in the longitudinal plane. It was also observed that the earliest motion was found at around 4 mm superficially from the aponeurosis in transverse plane. Meanwhile, not much variation in the proximal–distal direction was identified. While some interesting muscle contraction patterns were identified in this study, it is still necessary to refine the method for onset detection for a more accurate measurement and wide applications in the future.

### 4.2. Onset Time of Physiological Measurements and MVCs

This study detected a mean EMD of 73 ms, which is around half of that measured by Ubeda et al. at 10% MVC in the same muscle [24]. This difference could be partly caused by the difference in positioning of the electrode, which can lead to 40–60 ms of error in EMD [25]. Hence, the results obtained in this study can be considered to match those described in previous reports.

Unlike a previous study which observed that, for MVC levels lower than 10%, the EMD was longer when the MVC level was lower [24], the current study did not observe such an effect. It still remains unknown whether, at lower levels of MVCs, the ultrasound-detected onset would be dependent on MVC levels. Future studies are needed to look into this area for further clarification.

### 4.3. Accuracy and Reliability of Simultaneous Onset Measurement

The accuracy of onset detection method used for ultrafast ultrasound is subject to the effects of white noise. The amplitude of white noise is almost constant across the field of view in this study. Hence, the signal to noise ratio increased with the ultrasound intensity of each pixel. In other words, white noise tended to delay the onset detected for points with lower intensity. Indeed, we observed earlier onset in many but not all brighter areas. Since the noise in ultrasound image increases with depth, the onset detection in deep muscle is also less sensitive. Alternative onset detection methods such as Teager–Kaiser Energy Operator (TKEO) or Bayesian change point analysis may provide higher accuracy; however, the computation power and time required would be much higher [20]. Determining an appropriate onset detection method is a very important research topic for future study.

### 4.4. Insights for Future Ultrafast Ultrasound Onset Measurement

The presence of blood vessels in the field of view would give rise to periodic motions in the area around the vessels in ultrasound images. The motion would be shown as a baseline drift in the ultrasound intensity and affects the onset detection. Applying a high-pass filter could remove the baseline drift, but it would also induce unwanted artificial signal near the start of motion, making the detected onset earlier than the real onset. This problem is yet to be solved.

Since the sizes of human muscles are in general too large when compared to the ultrasound probe, muscle contraction could not be observed with the whole muscle in the field of view due to technological limitations. In addition to further advancing the technologies of ultrasound imaging modalities, this could also be addressed by conducting animal experiments in the future. One of the planned experiments involves recording rats’ gastrocnemius contraction during cortical electrical stimulation, of which the ultrasound recorded muscle motion can be compared with the EMG onset measured though intramuscular electrodes.

The current experimental setting cannot guarantee that the subjects contracted their TA before other muscle contractions. In some trials where the transverse view of muscle was imaged, the subjects contracted their extensor digitorum longus (the muscle lateral to TA) first in performing the requested motion. The motion of other muscles before TA would cause passive motion of TA, represented by a baseline drift in the data. The active and passive motion cannot be separated in this method at this moment.

Upon optimizing the experimental setup, the patterns of muscle onset in patients with muscle disorders are yet to be studied. Granata et al. showed that EMD in patients with spastic cerebral palsy is shorter than that in normal subjects in reflex response [6]. The amplitude of EMG signal normalized to MVC level is also higher in the patient group. It is expected that the pattern of onset time observed in ultrafast ultrasound would also be different in these patients. Ultrafast ultrasound provides a new way of studying how neuromuscular diseases affect muscle motions. Studying how these onset patterns differ in different neuromuscular diseases may offer new insights into the diseases and ways to improve patients’ motion.

## 5. Conclusions

A method for mapping spatial difference in the onset of muscle has been developed and evaluated. Preliminary results show that muscle onset starts at the aponeurosis. The level of MVC does not affect either the pattern of onset or the delay between the physiological measurements. The current onset detection method can be further improved to align with different study aims. It is expected that ultrafast ultrasound would provide a new way of studying how neuromuscular diseases affect muscle motion in the future.

## Figures and Tables

**Table 1:** Pairwise comparison between onset time at different muscle depths (along the depth of the image shown in Figure 3a, with D1 as the most superficial layer and D5 as the deepest layer. * *p* < 0.05, ** *p* < 0.01, *** *p* < 0.001, NA: not applicable, NS: no significant difference. *p*-value corrected using Bonferroni correction.

Depth	D1 (0–6 mm)	D2 (6–12 mm)	D3 (12–18 mm)	D4 (18–24 mm)	D5 (24–30 mm)	Order of Motion Onset
D1 (0–6 mm)	NA	t1 > t2 ***	t1 > t3 ***	t1 > t4 *	NS	4
D2 (6–12 mm)		NA	t2 < t3 **	t2 < t4 ***	t2 < t5 ***	1
D3 (12–18 mm)			NA	t3 < t4 ***	t3 < t5 ***	2
D4 (18–24 mm)				NA	t4 < t5 **	3
D5 (24–30 mm)					NA	4

**Table 2:** Pairwise comparison between onset time at different proximal–distal positions (along the width of the image shown in Figure 3a, with W1 as the most distal location and W5 as the most proximal location. * *p* < 0.05, NA: not applicable, NS: no significant difference. *p*-value corrected using Bonferroni correction.

Width	W1 (0–7.5 mm)	W2 (7.5–15 mm)	W3 (15–22.5 mm)	W4 (22.5–30 mm)	W5 (30–37.5 mm)	Order of Motion Onset
W1 (0–7.5 mm)	NA	t1 > t2 *	t1 > t3 *	t1 > t4 *	NS	NA
W2 (7.5–15 mm)		NA	NS	NS	NS	NA
W3 (15–22.5 mm)			NA	NS	NS	NA
W4 (22.5–30 mm)				NA	NS	NA
W5 (30–37.5 mm)					NA	NA

## References

[1] Esposito F., Limonta E., Cè E. (2010). Passive stretching effects on electromechanical delay and time course of recovery in human skeletal muscle: New insights from an electromyographic and mechanomyographic combined approach. Graefe’s Arch. Clin. Exp. Ophthalmol..

[2] Begovic H., Zhou G., Li T., Wang Y., Zheng Y. (2014). Detection of the electromechanical delay and its components during voluntary isometric contraction of the quadriceps femoris muscle. Front. Physiol..

[3] Begovic H., Zhou G., Schuster S., Zheng Y., Begovı H. (2016). The neuromotor effects of transverse friction massage. Man. Ther..

[4] Prutchi D. (1995). A high-resolution large array (HRLA) surface EMG system. Med. Eng. Phys..

[5] Oka H., Konishi Y., Kitawaki T., Ichihashi N., Yoshida M. Development of multichannel array transducer of displacement mechanical-myogram. Proceedings of the 2013 35th Annual International Conference of the IEEE Engineering in Medicine and Biology Society (EMBC).

[6] Granata K.P., Ikeda A.J., Abel M.F. (2000). Electromechanical delay and reflex response in spastic cerebral palsy. Arch. Phys. Med. Rehabilit..

[7] Karageanes S.J., Blackburn K., Vangelos Z.A. (2000). The Association of the Menstrual Cycle with the Laxity of the Anterior Cruciate Ligament in Adolescent Female Athletes. Clin. J. Sport Med..

[8] Stock M.S., Thompson B.J. (2016). Adipose tissue thickness does not affect the electromechanical delay. Physiol. Meas..

[9] Ibitoye M.O., Hamzaid N.A., Zuniga J.M., Wahab A.K.A. (2014). Mechanomyography and muscle function assessment: A review of current state and prospects. Clin. Biomech..

[10] Shi J., Zheng Y., Huang Q., Xin C. (2008). Continuous Monitoring of Sonomyography, Electromyography and Torque Generated by Normal Upper Arm Muscles During Isometric Contraction: Sonomyography Assessment for Arm Muscles. IEEE Trans. Biomed. Eng..

[11] Zheng Y., Chan M., Shi J., Chen X., Huang Q. (2006). Sonomyography: Monitoring morphological changes of forearm muscles in actions with the feasibility for the control of powered prosthesis. Med. Eng. Phys..

[12] Ma C.Z.-H., Ling Y.T., Shea Q.T.K., Wang L.-K., Wang X., Zheng Y. (2019). Towards Wearable Comprehensive Capture and Analysis of Skeletal Muscle Activity during Human Locomotion. Sensors.

[13] Grönlund C., Claesson K., Holtermann A. (2013). Imaging Two-Dimensional Mechanical Waves of Skeletal Muscle Contraction. Ultrasound Med. Biol..

[14] Vasseljen O., Dahl H.H., Mork P.J., Torp H.G. (2006). Muscle activity onset in the lumbar multifidus muscle recorded simultaneously by ultrasound imaging and intramuscular electromyography. Clin. Biomech..

[15] Vasseljen O., Fladmark A.M., Westad C., Torp H.G. (2009). Onset in abdominal muscles recorded simultaneously by ultrasound imaging and intramuscular electromyography. J. Electromyogr. Kinesiol..

[16] Hug F., Gallot T., Catheline S., Nordez A. (2011). Electromechanical delay in biceps brachii assessed by ultrafast ultrasonography. Muscle Nerve.

[17] Dieterich A.V., Botter A., Vieira T.M., Peolsson A., Petzke F., Davey P., Falla D. (2017). Spatial variation and inconsistency between estimates of onset of muscle activation from EMG and ultrasound. Sci. Rep..

[18] Deffieux T., Gennisson J.-L., Tanter M., Fink M., Nordez A. (2006). Ultrafast imaging of in vivo muscle contraction using ultrasound. Appl. Phys. Lett..

[19] Tweedell A.J., Haynes C., Tenan M. (2017). Computer-Based Algorithmic Determination of Muscle Movement Onset Using M-Mode Ultrasonography. Ultrasound Med. Biol..

[20] Tenan M., Tweedell A.J., Haynes C.A. (2017). Analysis of statistical and standard algorithms for detecting muscle onset with surface electromyography. PLoS ONE.

[21] Zhang J., Soangra R., Lockhart T.E. (2020). A Comparison of Denoising Methods in Onset Determination in Medial Gastrocnemius Muscle Activations during Stance. Science.

[22] Ortega-Cebrián S., Bagur-Cadafal C., Whiteley R., Navarro R., Monné-Guasch L., Girabent-Farrés M. (2020). Subacromial Impingment Syndrome does not alter muscle onset activation patterns during shoulder cardinal movement at different speed and load. Musculoskelet. Sci. Pr..

[23] Ortega-Cebrián S., Girabent-Farrés M., Whiteley R., Navarro R., Monné-Guasch L., Bagur-Calafat M.C. (2019). Shoulder muscle onset timing during clinical assessment movements is the same in elite handball players as non-athletes: Implications for clinical assessment. Phys. Ther. Sport.

[24] Úbeda A., Del Vecchio A., Sartori M., Puente S.T., Torres F., Azorín J.M., Farina D. Electromechanical delay in the tibialis anterior muscle during time-varying ankle dorsiflexion. Proceedings of the 2017 International Conference on Rehabilitation Robotics (ICORR).

[25] Hug F., Lacourpaille L., Nordez A. (2011). Electromechanical delay measured during a voluntary contraction should be interpreted with caution. Muscle Nerve.

